# Abnormal blood pressure circadian rhythms are relevant to cerebral infarction and Leukoaraiosis in hypertensive patients

**DOI:** 10.1186/s12883-020-1626-6

**Published:** 2020-01-28

**Authors:** Kang Yang, Xiaodong Zhu, Yulan Feng, Fanxia Shen, Jie Chen, Ningzhen Fu, Jialan Sun, Yi Fu

**Affiliations:** 1 Department of Neurology & Institute of Neurology, Rui Jin Hospital, School of Medicine, Shanghai Jiao Tong University, No.197, Rui Jin Er Road, Shanghai, 200025 China; 2 Department of Neurology, The First Hospital of Jiaxing, Zhejiang, 314000 China; 3 Department of Neurology, Minhang Hospital, Fudan University, Shanghai, 201100 China; 4 School of Medicine, Shanghai Jiao Tong University, Shanghai, 200025 China; 5 Department of Neurology, Pu Dong District Gonli hospital, Shanghai, 200120 China

**Keywords:** Blood pressure circadian rhythms, Acute cerebral infarction, Silent cerebral infarction, Leukoaraiosis

## Abstract

**Background:**

To investigate the relationships between blood pressure (BP) circadian rhythms and acute cerebral infarction (ACI), silent cerebral infarction (SCI) and the severity of leukoaraiosis in hypertensive patients.

**Methods:**

A retrospective case-control study was performed among hypertensive patients with 24-h ambulatory blood pressure monitoring (ABPM) and cranial magnetic resonance imaging (MRI).

**Results:**

A total of 1267 patients were enrolled. Lower nocturnal blood pressure (BP) decreases were observed in ACI patients than in controls (3.3% vs 8.2%, P<0.001). Reverse-dipper pattern (RD) and non-dipper pattern (ND) were found to be independent risk factors for ACI. In ACI patients, both RD and ND BP circadian rhythms were revealed to be independent risk factors for moderate-severe leukoaraiosis. In addition, in SCI patients, RD (OR = 1.7, 95% CI, 0.9–3.0; *P* = 0.047) or extreme-dipper pattern (ED) (OR = 2.9, 95% CI, 1.2–7.0; *P* = 0.015) were found to be independent risk factors for moderate-severe leukoaraiosis. Moreover, the greater the severity of leukoaraiosis was, the higher the ratio of abnormal BP circadian rhythms.

**Conclusion:**

RD and ND BP circadian rhythms might not only be relevant to the onset of ACI but also correlate with the severity of leukoaraiosis. Thus, when modulating BP with antihypertensive drugs, the BP circadian rhythms, and not merely the BP level, should warrant more attention.

## Background

Cerebral infarction (CI), one of the leading causes of global adult mortality and disability with a younger age of onset than before, threatens the health of human beings [1]. Hypertension is overwhelmingly believed by the public to be the main risk factor for CI [2]. Previous studies have illustrated that hypertensive patients with the loss of blood pressure (BP) circadian rhythms might be more susceptible to cardio-cerebral vascular incidents. Ambulatory blood pressure monitoring (ABPM), characterized by continuous BP measurement, provided not only the overall view of the 24-h BP but also the BP circadian pattern, especially nocturnal BP data, which could help predict prognosis [3].

In healthy populations, physiological 24-h BP circadian rhythms play a vital role in protecting the structure and function of vessels. The loss or attenuation of rhythms might lead to impairments in endothelial function, inflammatory reactions [4] and oxidative stress activations [5] and further induced or aggravated atherosclerosis. An increasing number of studies have revealed a close relationship between BP circadian rhythm changes and cerebral small vessel diseases (CSVDs) [6–8]. CSVD is the term generally utilized to describe clinical syndromes with cognitive dysfunctions and changes in neuroimaging and neuropathology. The imaging characteristics mainly consist of leukoaraiosis, lacunar infarction, cerebral microbleeds, lacuna, perivascular space enlargement and cerebral atrophy [9]. CSVDs have doubled the hazard of stroke, resulting in approximately 20% of stroke cases and leading to approximately 45% of dementia cases [10, 11], which have cast heavy economic burdens on society and attracted more attention from academia as well. Leukoaraiosis, initiated from an imaging concept, described the demyelinating disease of nerve conduction fibres caused by diffuse cerebral ischaemia with classical clinical symptoms such as chronic progressive dementia, slow thinking and processing and cognitive function decline. After a 2-year follow-up, a prospective study enrolled 96 hypertension patients with CI and indicated that the elevation of 24-h diurnal and nocturnal mean systolic BP (SBP) and diastolic BP (DBP) were closely related to cerebral microbleeds [12]. Another study recruited elderly individuals aged 75 to 89 years and declared a relevance between 24-h ambulatory blood pressure and the progression of leukoaraiosis with a 4-year follow-up [13]. However, the exact relationship between BP rhythms and leukoaraiosis remains under debate [14–18].

Due to the limitations of sample size and inconsistent conclusions in current articles targeting the relevance of BP circadian rhythms to acute cerebral infarction (ACI), silent cerebral infarction (SCI) and leukoaraiosis, no benefits have resulted from clinical disease treatment. Based on retrospective research, we investigated the exact relevance of BP circadian rhythms to the onset of ACI and SCI and the severity of leukoaraiosis.

## Methods

### Patient enrolment

The study was a retrospective case-control study recruiting 1267 hypertensive patients matching the inclusion criteria in Ruijin Hospital, affiliated with the School of Medicine, Shanghai Jiaotong University, and Minhang Hospital, affiliated with Fudan University, from January 2010 to November 2017. The inclusion criteria were as follows. First, adults were aged over 18 years old and had a hypertensive history. Hypertension were defined as 1) having a past hypertension history and 2) having persisting hypertension (SBP over 140 mmHg or DBP over 90 mmHg) in three separate BP measurements when hospitalized. In addition, evidence of end-stage organ impairments (e.g., hypertensive retinopathy, enlargement or hypertrophy of the left ventricle) was required. The second inclusion criterion pertained to acute cerebral infarction, silent cerebral infarction, and the control group. ACI was characterized as follows: a) sudden onset; b) focal neurological deficits such as hemiplegia, aphasia and others; and c) in cranial MRI examination, lesions showed high signal in the DWI sequence and were diagnosed as ACI by neurology physicians. Silent cerebral infarction was characterized by the following: patients without clear CI or transient ischaemic attack (TIA) history but lesions with high signal in the T2 WI or FLAIR sequences, low signal in the T1 WI sequence and low or equal signal in the DWI sequence. For the control group, the cranial MRI was normal. The third inclusion criterion was that ACI patients were examined with noninvasive ambulatory blood pressure monitoring (ABPM) 1 month after onset. The exclusion criteria were as follows: 1) Cardiogenic cerebral infarction (cardiogenic stroke is mostly caused by cardiogenic embolism shedding, whose pathogenesis differs from that of large atherosclerosis and small artery occlusion cerebral infarction); 2) Past stroke history; 3) Intracranial lesions such as bleeding or occupying lesions detected by cerebral CT or MRI; 4) Having severe respiratory diseases, coronary heart diseases, malignant tumours, metabolic diseases such as severe liver and kidney dysfunction or thyroid dysfunction; and 5) Having contraindications for MRI, pregnancy or refusal to participate in the study.

The data of the eligible patients were collected, comprising general demographic data (gender, age, duration of hypertension, history of past diseases (diabetes mellitus, for instance), smoking, alcohol intake), laboratory data (fasting blood glucose, creatinine, urea nitrogen, homocysteine, total cholesterol (TG), triglycerides (TC), high-density lipoprotein cholesterol (HDL-C), low-density lipoprotein cholesterol (LDL-C)), imaging information (sites of CI, existence and scores of leukoaraiosis) and ABPM data (24-h mean SBP, 24-h mean DBP, 24-h mean arterial pressure (MAP), diurnal mean SBP, diurnal mean DBP, diurnal MAP, nocturnal mean SBP, nocturnal mean DBP, nocturnal MAP and nocturnal BP decline rate (NBPDR)).

This study was approved by the ethics committee of Ruijin Hospital, School of Medicine, Shanghai Jiao Tong University.

### Examinations

All the subjects were examined with cranial MRI in the axial T1 WI and T2 WI sequences first and the DWI sequence next. Noninvasive ABPM was applied. The cuff was located on the left arm, and the measuring gap during the daytime (6:00 to 22:00) was 30 min, while it was 60 min at night. The SBP, DBP, mean BP and heart rate were recorded. The examination lasted over 24 h. The SCI and control group subjects were mainly from the hypertension clinic and cardiology clinic. For these subjects, ABPM was performed at home or at work. The daily activities of subjects were not limited; rather, they had a strict wake-up time of 6:00 and sleep time of 22:00. ABPM parameters were calculated and analysed with microcomputers, including diurnal mean BP and nocturnal mean BP, and further led to diurnal MAP, nocturnal MAP and NBPDR with formulas. The formulas were listed as follows: MAP = DBP + 1/3 *(SBP - DBP), NBPDR = (diurnal MAP - nocturnal MAP)/ diurnal MAP [19]. Dipper pattern (D) was defined as NBPDR ≥10 and < 20%, while extreme-dipper pattern (ED) was defined as ≥20%, non-dipper pattern (ND) was defined as ≥0 and < 10%, and reverse-dipper pattern (RD) was defined as < 0 [20, 21]. Among these rhythms, ED, ND and RD were deemed abnormal BP rhythms. Leukoaraiosis was referred to as a kind of imaging change that presented low density in CT scanning or high signal in T2 WI MRI at periventricular white matter or subcortical area (half oval central), which was subdivided into periventricular hyperintensities (PVH) and deep white matter hyperintensities (DWMH). According to the cerebral white matter impairment scale (Fazekas scale), leukoaraiosis was rated as none (score = 0), mild (score = 1), moderate (score = 2), or severe (score = 3) [ 22]. Thus, according to the scales above, leukoaraiosis was classified as no leukoaraiosis (level 0), mild leukoaraiosis (level 1) and moderate-severe leukoaraiosis (level 2). Two senior radiologists were assigned to read and record the imaging randomly. When divergence occurred, discussion, negotiation and consistency were achieved.

### Statistical analysis

SPSS18.0 and GraphPad Prism 5.0 were utilized for the statistical analysis. Baseline clinical data, laboratory data and BP data were assigned into three groups: the control group, the ACI group and the SCI group, and spontaneously in another way, into three groups: the non-leukoaraiosis group, the mild leukoaraiosis group and the moderate-severe leukoaraiosis group. The Kolmogorov-Smirnova normality test was applied for quantitative data. If the data were normally distributed, the data were expressed in the form of the mean ± SD, and comparisons between two groups were performed with the t-test, while comparisons among several groups were performed with the Dunnett test. The interquartile range (IQR) was used for data not fitting the normal distribution. The Mann-Whitney U test was used for comparisons between two groups, while the Kruskal-Wallis H test was used for comparisons among several groups. Frequencies and percentages were used to represent the count data with the χ^2^ test and Fisher’s exact test. Patients with dipper patterns were defined as 1 [reference]. With binary stepwise logistic regression analysis, the risk factors for CI, such as gender, age, smoking, diabetes mellitus, cholesterol, and low-density lipoprotein (LDL), were included in the model in the analysis of the relationship between 24-h ambulatory BP rhythms and the onset of ACI and SCI, the independent risk factors of moderate-severe leukoaraiosis and the relationship between 24-h ambulatory BP rhythms and moderate-severe leukoaraiosis. *P* < 0.05 was regarded as the sign of statistically significant differences.

## Results

### General data

**Fig. 1:**
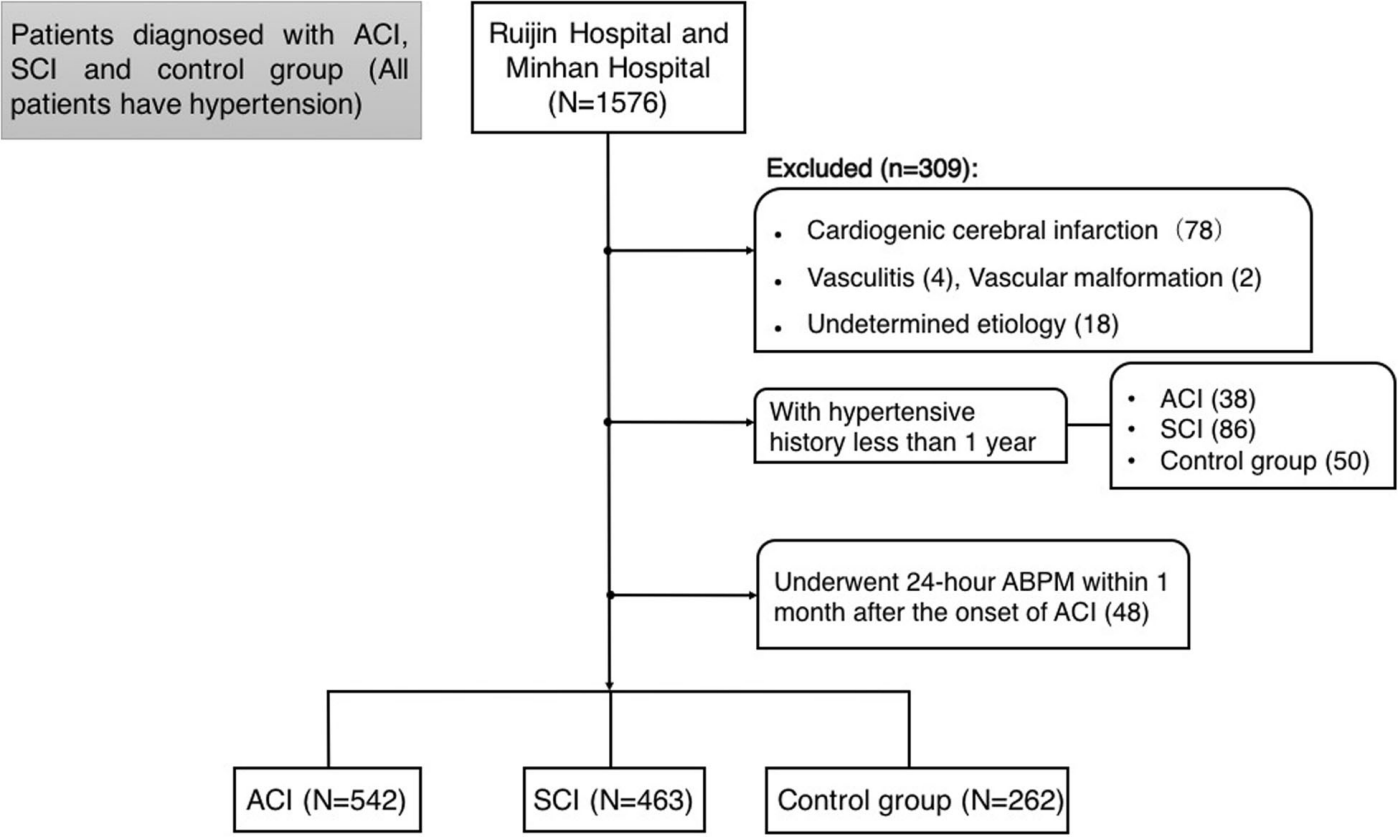
Flowchart of patient selection. ACI = acute cerebral infarction; SCI = silent cerebral infarction; ABPM = ambulatory blood pressure monitoring. Fifteen cases of ACI with hypertension less than 1 year and 24-h ABPM within 1 month of onset

According to the inclusion and exclusion criteria, 1267 hypertensive patients were eligible, with an average age of 65 ± 13 years old, among whom 702 subjects were male (55.4%). A flowchart of patient selection is provided in Fig. 1. All the patients had a history of hypertension, while 1128 (89.0%) of them took antihypertensive drugs. In total, 542 ACI patients, 463 SCI patients and 262 controls participated.

### Baseline data

**Fig. 2:**
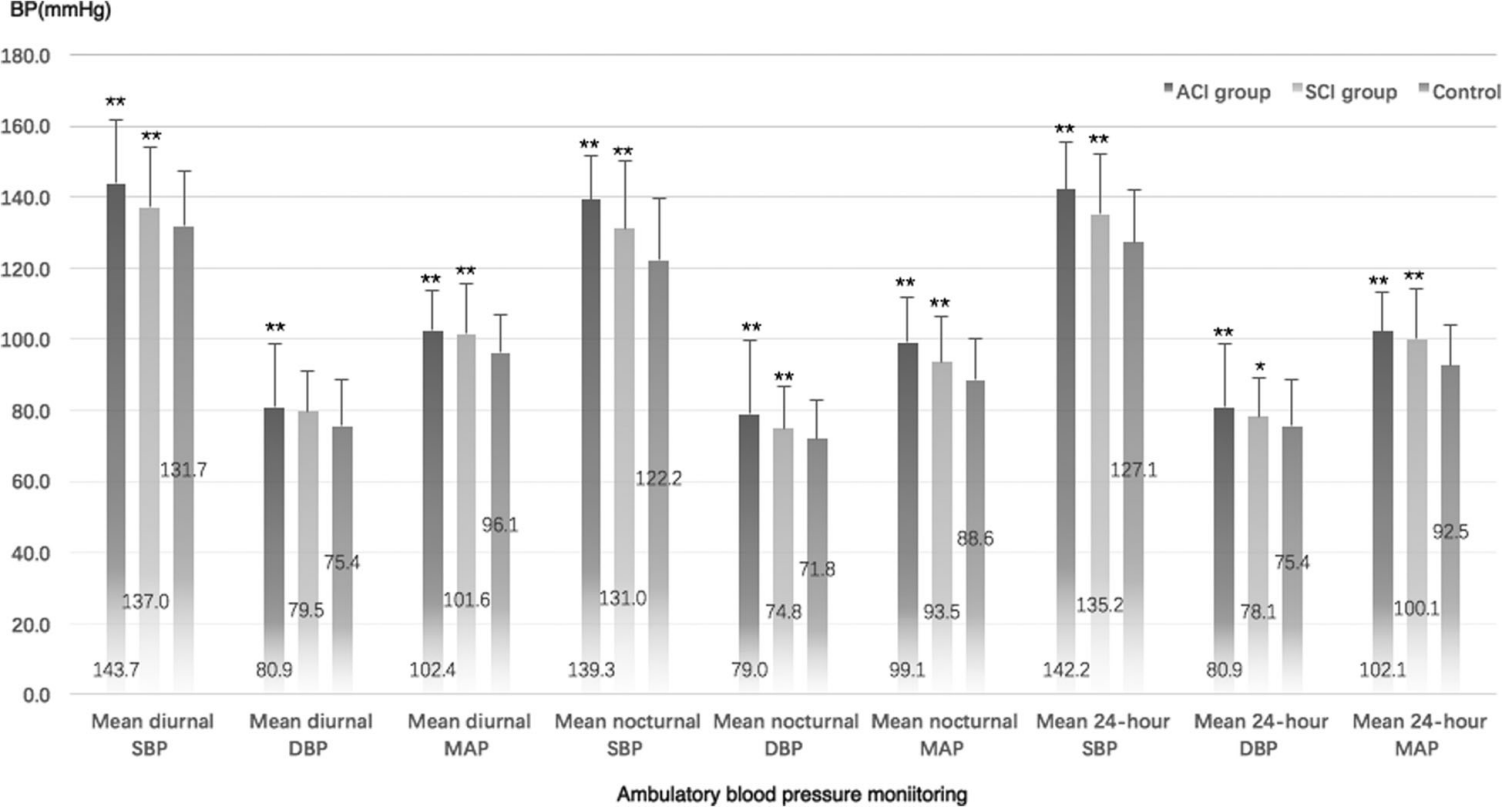
Ambulatory blood pressure monitoring in patients. Compared with the control group, ACI and SCI patients had significantly higher diurnal mean SBP, diurnal MAP, nocturnal mean SBP, nocturnal mean DBP, nocturnal MAP, 24-h mean SBP, 24-h mean DBP, and 24-h mean arterial pressure (MAP). BP = blood pressure; SBP = systolic blood pressure; DBP = diastolic blood pressure; MAP = mean arterial pressure. *p<0.05, **p<0.001

The characteristics and demographics of the ACI, SCI and control groups at baseline are listed in Table 1 and Fig. 2. Compared with controls, prominent elevations were observed for age, male ratio, smoking ratio, alcohol intake ratio, diabetes mellitus prevalence, hypertension duration, ratio of oral antiplatelet drug intake, leukoaraiosis prevalence, 24-h mean SBP, 24-h mean DBP, 24-h mean arterial pressure (MAP), diurnal mean SBP, diurnal MAP, nocturnal mean SBP, nocturnal mean DBP and nocturnal MAP in ACI and SCI patients, with significantly less NBPDRs (3.3% vs 7.9%, *P*<0.001) in ACI patients and no significant difference in SCI patients.

**Table 1:** Characteristics and demographics of ACI, SCI and control groups at baseline

	ACI group (*N* = 542)	SCI group (*N* = 463)	Control (*N* = 262)	*P* 1	*P* 2
Age	66.8 ± 12.3	65.9 ± 12.8	57.8 ± 10.2	<0.001	<0.001
Gender, Male	342 (63.1%)	255 (55.1%)	105 (40.1%)	<0.001	<0.001
Smoking, Yes	195 (36.0%)	135 (29.2%)	35 (13.4%)	<0.001	<0.001
Alcohol, Yes	116 (21.4%)	90 (19.4%)	24 (9.2%)	<0.001	<0.001
Diabetes mellitus, Yes	166 (30.6%)	118 (25.5%)	31 (11.8%)	<0.001	<0.001
Hypertension history (yrs)	10.0 (5.0–20.0)	10.0 (5.0–20.0)	5.0 (2.0–10.0)	<0.001	<0.001
Hypertension family history, Yes	46 (8.5%)	202 (43.6%)	79 (30.2%)	<0.001	<0.001
Oral medicine, Yes					
Antihypertension drugs	480 (88.6%)	396 (85.5%)	252 (96.2%)	<0.001	<0.001
Antiplatelet drugs	290 (53.5%)	152 (32.8%)	51 (19.5%)	<0.001	<0.001
Laboratory data					
Cholesterol, mmol/L	4.7 ± 1.1	4.6 ± 1.0	4.6 ± 1.1	0.583	0.894
Triglyceride, mmol/L	1.8 ± 1.2	1.8 ± 1.1	1.8 ± 1.7	0.941	0.645
HDL, mmol/L	1.1 ± 0.3	1.2 ± 0.7	1.3 ± 0.9	<0.001	0.013
LDL, mmol/L	2.9 ± 0.9	2.9 ± 1.0	2.9 ± 0.9	0.531	0.265
FBG, mmol/L	6.1 ± 2.3	5.5 ± 1.5	5.4 ± 1.3	<0.001	0.542
Urea nitrogen, mmol/L	4.9 (4.1–5.9)	5.1 (4.2–6.3)	5.2 (4.2–6.8)	<0.001	<0.001
Creatinine, μmol/L	72.0 (61.0–88.3)	73.0 (58.0–86.3)	61.0 (49.0–77.0)	<0.001	<0.001
Homocysteine, mmol/L	14.6 ± 11.1	15.2 ± 11.4	12.7 ± 5.2	0.206	0.109
TOAST					
AS	429 (79.2%)	–	–		
LS	113 (20.8%)	–	–		
Leukoaraiosis, Yes	448 (82.7%)	370 (79.9%)	–		
Leukoaraiosis, PVWM					
Non	98 (18.1%)	95 (20.5%)	–		
Mild	122 (22.5%)	139 (30.0%)	–		
Moderate	169 (31.2%)	130 (28.1%)	–		
Severe	153 (28.2%)	99 (21.4%)	–		
Leukoaraiosis, DWM			–		
Non	120 (22.1%)	123 (26.6%)	–		
Mild	130 (24.0%)	139 (30.0%)	–		
Moderate	117 (21.6%)	97 (21.0%)	–		
Severe	175 (32.3%)	104 (22.5%)	–		
Moderate-Severe leukoaraiosis	345 (63.7%)	244 (52.7%)	–		
NBPDR, %	3.3 (−1.8–8.1)	8.2 (1.8–14.2)	7.9 (2.7–13.8)	<0.001	0.638

*Abbreviations*: *ACI* acute cerebral infarction, *SCI* silent cerebral infarction, *HDL* high-density lipoprotein cholesterol, *LDL* low-density lipoprotein cholesterol, *FBG* fasting blood glucose, *TOAST* Trial Ord 10,172 in acute stroke treatment, *AS* atherothrombotic stroke, *LS* lacunar stroke, *PVWM* periventricular hyperintensities, *DWM* deep white matter, *NBPDR* Nocturnal blood pressure drop rate. *P*1 and *P*2 refer to the comparisons of ACI group with control group and SCI group with control group separately

^a^Results are expressed as number (column %), mean ± SD, or median (interquartile range), as appropriate

### Relevance between BP circadian rhythms and ACI or SCI

**Fig. 3:**
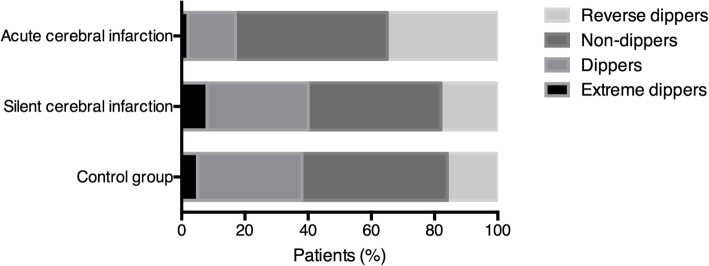
BP circadian rhythm distribution in the ACI, SCI and control groups. In ACI patients, SCI patients and the control group, the respective number of individuals with each pattern of BP was as follows: reverse-dipper pattern: 186 (34.3%), 84 (18.1%), and 40 (15.3%); non-dipper pattern: 261 (48.2%), 193 (41.7%), and 123 (46.9%); dipper pattern: 83 (15.3%), 149 (32.2%), and 86 (32.8%); and extreme-dipper pattern: 12 (2.2%), 37 (8.0%), and 13 (5.0%)

The BP circadian rhythms in ACI, SCI and control patients are shown in Fig. 3. We subclassified ACI into atherothrombotic stroke and lacunar stroke and divided the ACI group into three subgroups: atherothrombotic stroke, lacunar stroke and both. With univariate analysis, increasing risks of ACI in all patients with RD and ND BP rhythms were observed (Table 2). Therefore, we further performed multivariate analysis and established a binary stepwise logistic regression model. The risk factors related to ACI were all included in the model, and it is no accident that RD and ND BP rhythms were independent risk factors for ACI. For SCI, no clear influence of BP circadian rhythms was discovered.

**Table 2:** Relevance between BP circadian rhythms and ACI or SCI groups

	Dipper pattern	Reverse-dipper pattern HR (95%CI)	Non-dipper pattern HR (95%CI)	Extreme-dipper pattern HR (95%CI)
ACI group				
AS group				
Unadjusted	1 [Reference]	4.51 (2.8–7.2)**	1.90 (1.3–2.8)**	0.92 (0.4–2.2)
Adjusted	1 [Reference]	3.40 (2.0–5.8)**	1.78 (1.1–2.8)*	1.09 (0.4–3.0)
LS group				
Unadjusted	1 [Reference]	6.84 (3.2–14.8)**	4.13 (2.1–8.3)**	1.20 (0.2–6.1)
Adjusted	1 [Reference]	4.36 (1.8–10.6)**	3.75 (1.7–8.2)**	2.40 (0.4–14.8)
AS or LS group				
Unadjusted	1 [Reference]	4.82 (3.1–7.6)**	2.20 (1.5–3.2)**	0.92 (0.4–2.2)
Adjusted	1 [Reference]	3.50 (2.1–5.9)**	2.00 (1.3–3.0)**	1.16 (0.4–3.1)
SCI group				
Unadjusted	1 [Reference]	1.21 (0.8–1.9)	0.91 (0.6–1.3)	1.64 (0.8–3.3)
Adjusted	1 [Reference]	0.82 (0.5–1.4)	0.91 (0.6–1.3)	1.56 (0.7–3.3)

Analysis focused on the comparisons as ACI group with control group and SCI group with control group separately, with the multivariate analysis adjustment on gender, age, smoking, diabetes, cholesterol, LDL and so on. *AS* atherothrombotic stroke, *LS* lacunar stroke, *HR* hazard ratio, *CI* confidence interval. (* *P*<0.05, ** *P*<0.001)

### Association between leukoaraiosis severity and BP level together with BP circadian rhythms

In ACI and SCI patients, comparisons were processed according to different leukoaraiosis severities, BP levels and BP rhythm types separately (Table 3). We divided ACI patients into three groups, including 96 cases of no leukoaraiosis, 101 cases of mild leukoaraiosis and 345 cases of moderate-severe leukoaraiosis; the abnormal BP circadian rhythms in each group were 79 (82.3%), 84 (83.2%) and 296 (85.8%), respectively. In SCI patients, there were 91 cases of no leukoaraiosis, 128 cases of mild leukoaraiosis and 244 cases of moderate-severe leukoaraiosis; the abnormal circadian rhythm of each group was 51 (56%), 88 (68.8%) and 175 (71.7%), respectively. Along with the increasing severity of leukoaraiosis, the ratio with abnormal BP rhythms increased. Then, we embarked on the analysis of the relationship between leukoaraiosis severity and different BP rhythms (Table 4). In mild leukoaraiosis patients, ACI patients with RD and ND rhythms more easily suffered mild leukoaraiosis than those with D rhythms. However, in multivariate analysis, no significant correlation was observed. No correlation was discovered between BP rhythms and mild leukoaraiosis in SCI patients. For moderate-severe leukoaraiosis, regardless of univariate analysis or multivariate analysis, RD and ND BP rhythms were independent risk factors for moderate-severe leukoaraiosis in ACI patients. With respect to SCI patients, patients with RD and ED BP rhythms had a higher risk of moderate-severe leukoaraiosis than those with D rhythm. Multivariate analysis also revealed that RD (OR = 1.7, 95% CI, 0.9–3.0; *P* = 0.047) and ED pattern (OR = 2.9, 95% CI, 1.2–7.0; *P* = 0.015) BP rhythms were independent risk factors for moderate-severe leukoaraiosis in SCI patients.

**Table 3:** Comparison between leukoaraiosis severity and BP level together with different BP rhythms

	ACI group (N = 542)				SCI group (N = 463)	
	Mild leukoaraiosis	Moderate-severe leukoaraiosis	Non leukoaraiosis	Mild leukoaraiosis	Moderate-severe leukoaraiosis	Non leukoaraiosis
	(*N* = 101)	(*N* = 345)	(*N* = 96)	(*N* = 128)	(*N* = 244)	(*N* = 91)
BP, mmHg						
Mean diurnal SBP	141.6 ± 16.2	144.7 ± 18.3	142.1 ± 18.5	134.6 ± 14.4	139.5 ± 18.6**	133.8 ± 14.0
Mean diurnal DBP	83.8 ± 11.6	80.3 ± 11.5**	84.8 ± 10.5	79.3 ± 9.9	78.7 ± 12.0*	82.1 ± 11.0
Mean diurnal MAP	103.1 ± 11.8	101.8 ± 12.2	103.9 ± 11.7	99.6 ± 11.0	103.4 ± 15.8*	99.6 ± 10.6
Mean nocturnal SBP	134.6 ± 18.9	141.4 ± 20.7	136.7 ± 20.9	126.6 ± 17.1	136.4 ± 20.4**	122.6 ± 14.9
Mean nocturnal DBP	79.9 ± 12.7	78.0 ± 12.3**	81.5 ± 12.2	73.7 ± 10.6	75.4 ± 13.0	74.4 ± 10.4
Mean nocturnal MAP	98.1 ± 13.4	99.1 ± 13.5	99.9 ± 13.7	91.3 ± 11.3	95.8 ± 13.9**	90.5 ± 10.9
Mean 24-h SBP	139.6 ± 16.1	143.4 ± 18.1	140.3 ± 18.7	132.2 ± 14.6	138.5 ± 18.1**	130.4 ± 13.6
Mean 24-h DBP	82.7 ± 11.4	79.6 ± 11.1**	83.8 ± 10.6	77.6 ± 9.8	77.7 ± 11.8	79.7 ± 10.5
Mean 24-h MAP	101.9 ± 12.2	101.9 ± 14.3	102.5 ± 11.8	97.8 ± 11.2	102.6 ± 15.8**	96.8 ± 10.8
NBPDR, %	4.2 (−0.7–9.6)	2.9 (− 2.5–7.3)	3.7 (−1.1–8.2)	8.1 (1.9–14.4)	6.7 (0.7–14.1)*	9.2 (5.1–13.7)
BP circadian rhythms classification						
Reverse-dipper	27 (26.7%)	125 (36.2%)	34 (35.4%)	19 (14.8%)	58 (23.8%)	7 (7.7%)
Non-dipper	52 (51.5%)	165 (47.8%)	44 (45.8%)	58 (45.3%)	94 (38.5%)	41 (45.1%)
Dipper	17 (16.8%)	49 (14.2%)	17 (17.7%)	40 (31.3%)	69 (28.3%)	40 (44.0%)
Extreme-dipper	5 (5.0%)	6 (1.7%)	1 (1.0%)	11 (8.6%)	23 (9.4%)	3 (3.3%)

*P<0.05, ** *P*<0.001

**Table 4:** Relevance between leukoaraiosis severity and different BP circadian rhythms

	Relevance between mild leukoaraiosis and different BP circadian rhythms				Relevance between moderate-severe leukoaraiosis and different BP circadian rhythms			
	ACI group		SCI group		ACI group		SCI group	
	OR (95% CI)	*P* value	OR (95% CI)	*P* value	OR (95% CI)	*P* value	OR (95% CI)	*P* value
Unadjusted								
Dipper	1 [Reference]	1 [Reference]	1 [Reference]	1 [Reference]	1 [Reference]	1 [Reference]	1 [Reference]	1 [Reference]
Reverse-dipper	2.2 (1.1–4.3)	0.022	1.3 (0.7–2.4)	0.46	3.6 (2.3–5.5)	<0.001	2.3 (1.4–3.7)	0.001
Non-dipper	1.9 (1.0–3.4)	0.038	1.1 (0.7–1.8)	0.649	2.0 (1.4–3.1)	<0.001	1.0 (0.7–1.5)	0.818
Extreme-dipper	2.2 (0.7–6.8)	0.186	2.2 (0.9–5.0)	0.073	0.9 (0.3–2.5)	0.84	2.6 (1.3–5.3)	0.007
Adjusted								
Dipper	1 [Reference]	1 [Reference]	1 [Reference]	1 [Reference]	1 [Reference]	1 [Reference]	1 [Reference]	1 [Reference]
Reverse-dipper	1.8 (0.9–3.6)	0.117	1.0 (0.5–2.0)	0.986	2.1 (1.2–3.5)	0.007	1.7 (0.9–3.0)	0.047
Non-dipper	1.7 (0.9–3.2)	0.106	1.2 (0.7–1.9)	0.573	1.8 (1.1–3.0)	0.012	1.1 (0.7–1.8)	0.578
Extreme-dipper	2.9 (0.9–9.6)	0.082	2.1 (0.8–5.1)	0.117	0.8 (0.2–2.9)	0.786	2.9 (1.2–7.0)	0.015

Multivariate analysis adjusted the factors such as gender, age, smoking, diabetes, cholesterol, LDL and so on

## Discussion

We drew the following conclusions from our results. First, the NBPDR of ACI patients was obviously lower than that of controls. RD pattern and ND pattern BP rhythms were found to be independent risk factors for ACI, including atherothrombotic stroke and lacunar stroke, with binary stepwise logistic regression analysis. No distinct difference in NBPDR existed between the SCI and control groups. Second, the more severe the leukoaraiosis is, the higher the ratio of abnormal BP rhythms. Third, RD and ND BP rhythms were independent risk factors for moderate-severe leukoaraiosis in ACI patients. Fourth, RD and ED BP rhythms were independent risk factors for moderate-severe leukoaraiosis in SCI patients.

In recent years, an increasing number of studies have shown not only that CI is related to BP levels, but also that elevated BP variances play a role as an independent predictive factor [23]. A study with 362 hypertensive patients noted that RD pattern BP was an independent risk factor for ACI [24]. Our former study also observed the probable relationship between RD BP circadian rhythm and the onset of spontaneous cerebral haemorrhage [25]. Both a cross-sectional study and a longitudinal study showed an increase in the incidence of cardiovascular disease and its increased related mortality and disability in ND BP patients [26, 27]. This time, we also observed that the NBPDR of ACI patients was lower than that in controls. In addition, RD and ND abnormal BP rhythms were independent risk factors for ACI. The BP of hypertensive patients with RD and ND BP rhythms merely decreased slightly at night compared with diurnal BP or even increased, which kept the cerebral vessels in a hypertension load state at night and further aggravated endothelial impairment, causing an inflammation reaction and accelerated progression of atherosclerosis. Given that the vessels were stenosed, the cerebral blood flow regulation function weakened, and individuals were more susceptible to stroke [28]. Therefore, abnormal BP rhythms (especially RD and ND) require more attention, and adjusting antihypertensive drug intake according to BP rhythms could greatly benefit hypertensive patients.

Leukoaraiosis could increase the risk of stroke, lead to a poor prognosis [29], and be closely related to decreases in cognitive function [30] and dyskinesia [31]. Age was positively correlated with the incidence of leukoaraiosis. Moreover, most studies based on the community illustrated that hypertension was correlated with not only the onset of leukoaraiosis but also its progression [32–36]. Arteriole atherosclerosis was regarded as the main cause in related studies [37]. Nevertheless, conflicts exist regarding the association between BP variation and leukoaraiosis. Dickie et al. [18] believed no association existed. Though with a large sample size and multicentre participation, there were still some limitations, including some researchers evaluating leukoaraiosis with CT scans, the sensitivity of which was significantly worse than that of MRI. Other studies indicated that BP variances were correlated with leukoaraiosis and even its progression [16, 38]. These different conclusions might be ascribed to the different enrolled populations (e.g., age, risk factors for cardiovascular diseases, past stroke history). Additionally, some of the inconsistencies may be attributed to the different leukoaraiosis evaluation methods. In our study, BP variances were found to be relevant to leukoaraiosis. In addition, the more severe the leukoaraiosis, the higher the ratio of abnormal BP rhythms. RD and ND BP rhythms were independent risk factors for moderate-severe leukoaraiosis in ACI patients, while RD and ED BP rhythms were independent risk factors for moderate-severe leukoaraiosis in SCI patients.

Similar to many past studies, the lower the NBPDR (RD, for example) was, the worse the leukoaraiosis would be [39]. Hypertensive patients, especially chronic hypertensive patients, with RD BP suffered an increase in BP at night, which induced inner wall thickening and hyaline degeneration of intracranial arterioles and deep perforating arteries. Due to the lack of deep perforating arteries and their collateral circulation, cerebral white matter is injured, and leukoaraiosis then occurs [40]. In addition, our research found that in SCI patients, not only those with the RD BP rhythm but also patients with ED BP rhythm endured a higher risk of moderate-severe leukoaraiosis. In addition, both were independent risk factors. That is, regardless of whether nocturnal BP increased or decreased too much, SCI patients suffered more hazards of moderate-severe leukoaraiosis. The relationship between hypotension and leukoaraiosis might be interrelated with the damage to cerebral self-regulation function [41, 42], which requires special attention. Fortunately, the population with ED BP rhythm only comprises a small proportion of hypertensive patients. The Rotterdam Scan Study (RSS) also found that impairment of the cerebral vasomotor response was correlated with leukoaraiosis [43]. When systemic BP decreased, the cerebral vessels with impaired vasomotor response may not have been able to enlarge to increase cerebral blood flow, which may have led to cerebral haemodynamic ischaemic dysfunction. In the recent Vitamins To Prevent Stroke (VITATOPS) research subgroup analysis, lower baseline DBP could help predict cognitive function decline in severe leukoaraiosis patients [44]. Therefore, BP with normal rhythms was essential for the protection of vessel structure and function. Several community-based studies found that maintaining BP at a certain level effectively could lower the incidence of leukoaraiosis [45]. As a controllable risk factor, the recognition of the relationship between BP ambulatory changes and leukoaraiosis helped control BP properly at an early stage and delayed the onset and progression of leukoaraiosis.

All cases of leukoaraiosis were evaluated by MRI. However, the following deficiencies still existed in our study. First, all of our enrolled patients should be examined with MRI and 24-h ABPM, which could exclude patients with slight or severe symptoms due to the lack of MRI. The strict inclusion criteria caused certain selection bias. Second, most patients took antihypertension drugs when examined with ABPM, which might have influenced our results. Finally, owing to the limited ratio of the population with ED BP rhythm among hypertension patients, the findings regarding ED BP rhythm were not sufficient.

## Conclusion

Overall, we considered that patients with RD and ND BP rhythms suffered a higher risk of ACI. Abnormal BP rhythms were closely correlated with the severity of leukoaraiosis. Additionally, we can infer that we should perform close follow-up of hypertensive patients with abnormal BP rhythms, given that this is a controllable risk factor, and consider BP rhythm regulation when formulating antihypertension drug intake plans, which have certain clinical significance in preventing CI and cognitive behavioural disorders.

## Data Availability

The datasets used and/or analysed during the current study are available from the corresponding author on reasonable request.
